# Comparative toxicity of imidacloprid and thiacloprid to different species of soil invertebrates

**DOI:** 10.1007/s10646-017-1790-7

**Published:** 2017-03-23

**Authors:** Cláudia de Lima e Silva, Nicola Brennan, Jitske M. Brouwer, Daniël Commandeur, Rudo A. Verweij, Cornelis A. M. van Gestel

**Affiliations:** Department of Ecological Science, Faculty of Earth and Life Sciences, Vrije Universiteit, De Boelelaan 1085, 1081 HV Amsterdam, The Netherlands

**Keywords:** Neonicotinoids, Earthworms, Enchytraeids, Collembola, Isopods, Oribatid mites

## Abstract

Neonicotinoid insecticides have come under increasing scrutiny for their impact on non-target organisms, especially pollinators. The current scientific literature is mainly focused on the impact of these insecticides on pollinators and some aquatic insects, leaving a knowledge gap concerning soil invertebrates. This study aimed at filling this gap, by determining the toxicity of imidacloprid and thiacloprid to five species of soil invertebrates: earthworms (*Eisenia andrei*), enchytraeids (*Enchytraeus crypticus*), Collembola (*Folsomia candida*), oribatid mites (*Oppia nitens*) and isopods (*Porcellio scaber*). Tests focused on survival and reproduction or growth, after 3–5 weeks exposure in natural LUFA 2.2 standard soil. Imidacloprid was more toxic than thiacloprid for all species tested. *F. candida* and *E. andrei* were the most sensitive species, with LC_50_s of 0.20–0.62 and 0.77 mg/kg dry soil for imidacloprid and 2.7–3.9 and 7.1 mg/kg dry soil for thiacloprid. EC_50_s for effects on the reproduction of *F. candida* and *E. andrei* were 0.097–0.30 and 0.39 mg/kg dry soil for imidacloprid and 1.7–2.4 and 0.44 mg/kg dry soil for thiacloprid. The least sensitive species were *O. nitens* and *P. scaber*. Enchytraeids were a factor of 5–40 less sensitive than the taxonomically related earthworm, depending on the endpoint considered. Although not all the species showed high sensitivity to the neonicotinoids tested, these results raise awareness about the effects these insecticides can have on non-target soil invertebrates.

## Introduction

Neonicotinoids are neuroactive insecticides which act on nicotinic acetylcholine receptors (nAChR) on the post synaptic membrane, disrupting neural transmission in the central nervous system of insects (Tomizawa and Casida 2003, 2005). This can lead to sub-lethal effects, such as paralysis or even the death of the animal (Simon-Delso et al. 2015). These compounds are the biggest selling and most widely used group of insecticides, due to their effective action against a broad spectrum of plant-sucking insects like aphids, leafhoppers and whiteflies (Jeschke et al. 2011). Neonicotinoids have an extensive application range, with imidacloprid being the most used one (Nauen et al. 2008).

In commercial agriculture, a major proportion of neonicotinoids is applied in the form of seed treatment which theoretically provides accurate targeting through systemic action in the plant (Douglas and Tooker 2015), reducing drift of the compound into the ecosystem (EASAC 2015). Apart from seed dressing, these insecticides can also be used as soil treatments and foliar spray (Jeschke et al. 2011), soil drench, irrigation water and trunk injection (Goulson 2013). Due to their broad application methods and persistence in soil, different species of non-target organisms can be affected, involving different routes of exposure (pore water, food or skin contact).

Although seed dressing can reduce the risk of drift, this preventive type of application has been criticised in light of the toxicity risk to non-target invertebrates, due to the persistence of the insecticides in soil and its inconsistency with the principles of Integrated Pest Management (Goulson 2013; Hallmann et al. 2014; Iwasa et al. 2004; Kindemba 2009). Sur and Stork (2003) reported that plant uptake of neonicotinoids from seed dressing is limited to just 1.6% of the applied dosage in the target crop, leaving around 98% of the compound in the soil (Goulson 2013). This suggests that there will be exposure of soil invertebrates, which are functionally essential in soil nutrient cycling and useful bio-indicators (Van Gestel 2012). In 2013, the European Union, through the Regulation n.485 (EU 2013), disciplined the use and applications of three neonicotinoids within its member states (clothianidin, thiametoxam, and imidacloprid) pending more research on their toxicity.

Iwasa et al. (2004) studied the toxicity of imidacloprid, a nitro-substituted compound, and thiacloprid (N-cyanoamidine) to honey bees and found an LD_50_ for imidacloprid of 0.018 µg per bee, while for thiacloprid it was around three orders of magnitude higher (LD_50_ = 14.6 µg per bee). The large difference in toxicity of these compounds is thought to be due to the faster rate of metabolism of the cyano group compared to the nitroguanidine group, which results in detoxification in bees (Iwasa et al. 2004). Nevertheless, the difference is remarkable when considering that both insecticides are applied at more or less the same rates, being 75 g/ha for imidacloprid and 62.5 g/ha for thiacloprid (Pisa et al. 2015). It is still unknown how this difference in acute toxicity to honeybees translates to differences in the effects of these two neonicotinoids on other organisms, neither is known which are the differences in their long-term or chronic toxicity. Most of the research concerning these insecticides is focused on toxicity to pollinators and aquatic insects, neglecting their impact on soil invertebrates. Although some toxicity data is available, mainly for earthworms, there is a general lack of knowledge about the influence on other non-target soil organisms (EASAC 2015; Pelosi et al. 2014; Pisa et al. 2015).

The aim of this study was to investigate the toxicity of imidacloprid and thiacloprid to five species of soil invertebrates—the collembolan *Folsomia candida*, the oribatid mite *Oppia niten*s, the isopod *Porcellio scaber*; and two oligochaetes: the earthworm *Eisenia andrei* and the enchytraeid *Enchytraeus crypticus*. These species are standard bio-indicators representative of the soil community with distinct life histories and body plans, belonging to different functional groups, with different exposure routes—pore water, food or direct contact with the soil, some living deeper in the soil and others living at the soil surface (Løkke and van Gestel 1998). Two key questions were targeted: 1. How sensitive are the different species of soil invertebrates to imidacloprid and thiacloprid, e.g., at which concentrations do these compounds cause toxicity and which species are most sensitive? 2. Is there a difference in toxicity of imidacloprid and thiacloprid, and does this difference depend on the endpoint chosen (e.g., survival, reproduction)?

## Materials and methods

### Test soil and treatments

All tests were performed using natural standard LUFA 2.2 soil (Lufa Speyer, Germany), having approximately 1.6% organic carbon, water holding capacity (WHC) of 45%, and soil pH (0.01 M CaCl_2_) measured in a preliminary test, ranging between 5.03 and 5.87. Pure imidacloprid and thiacloprid (purity 98%) were kindly provided by Bayer CropScience, Monheim, Germany. Since especially thiacloprid (log K_ow_ 1.26) was a bit hard to dissolve in water at higher concentrations, stock solutions were prepared in acetone (97%), with the exception of the water dilutions used for *F. candida* reproduction tests.

For acetone spiking, small portions of dry LUFA 2.2 soil (5 or 25 g) were spiked with 5 or 20 ml acetone stock solution in a glass jar (100 ml). The jars were closed with a plastic screw top lid and incubated overnight to allow for equilibration under a fume cupboard. Following equilibration and evaporation of the acetone, the spiked soils were mixed with the rest of the untreated Lufa 2.2 soil needed to achieve the desired concentration and moistened with distilled water to 50% WHC.

Test concentrations of imidacloprid and thiacloprid (Table S1 in the Supporting Information) were based on the results of range-finding tests for *E. crypticus*, *F. candida*, *O. nitens* and *P. scaber*, and on literature data on their toxicity to *E. andrei* and other species (Alves et al. 2013; Drobne et al. 2008, Capowiez et al. 2005; Luo et al. 1999; Zang et al. 2000). Five concentrations were used in the range-finding tests for both neonicotinoids: 0.01–0.1–1.0–10.0–100 mg/kg dry weight with one replicate conducted per treatment. *O. nitens* proved to be relatively insensitive in the range finding test and for this reason just two concentrations were followed up in the definitive one. Nominal exposure concentrations for the definitive tests ranged from 0.1–10 mg/kg dry soil for earthworms, 0.12–30 mg/kg dry soil for enchytraeids; 1–32 mg/kg dry soil for isopods; and 100 and 1000 mg/kg dry soil for mites (Table S1). With the exception of the second reproduction test performed with *F. candida*, two controls were used: one with and one without the addition of acetone. If possible, tests with both compounds were run simultaneously for a species, always using animals from the same culture or batch of age-synchronized animals.

For determining the toxicity of imidacloprid to springtails two sets of tests were performed. The concentrations used in the first were: 0.001–1.0 mg/kg dry soil, and for the second test: 0.03–1.0 mg/kg dry soil. For thiacloprid concentrations used were 0.01–10 mg/kg dry soil (Table S1). The first test with thiacloprid showed large variations, and therefore was not considered. Since this might be due to acetone spiking, the second test with imidacloprid and thiacloprid also compared spiking the compounds dissolved in water and in acetone. Tests with soil spiked with imidacloprid or thiacloprid in water and acetone started on the same day; imidacloprid and thiacloprid tests started with 1 day difference between them. In both cases, animals used were from the same age-synchronized cultures.

### Toxicity tests

Earthworms (*Eisenia andrei*) were obtained from a laboratory culture at the Department of Ecological Science, Vrije Universiteit, Amsterdam. Following OECD Guideline 222 (OECD 2004a), ten adult earthworms were exposed in 800 ml glass jars with approximately 600 g moist soil, having four replicates per concentration and control. Adults were taken from a 2-month age-synchronised culture and acclimatized for 24 h in clean LUFA 2.2 soil moistened at 50% of its WHC. Ten animals were randomly selected, washed, blotted dry on tissue paper and weighed and introduced into each test jar. To assess individual worms masses and its variation, for each test jar two earthworms were also weighed individually. Average worm masses (±SE) were 545 ± 15 and 552 ± 14 for the tests with imidacloprid and thiacloprid, respectively. Test jars were covered with opaque metal lids, loosely attached to allow for some aeration. To feed the earthworms, on the second day of exposure 5 g horse manure moistened with approximately 5 ml of distilled water was placed in a small hole made in the soil. On day 28, adults from each jar were counted, washed, blotted dry on tissue paper and weighed. After removing the adults, the soil including cocoons was carefully returned into the test container and incubated again in the climate room for another 28 days. After this period all test jars were placed in a water bath (Julabo TW12) at 60 °C to extract and count the juveniles emerging from the soil.

*Enchytraeus crypticus* was exposed for 21 days using an adaption of the OECD Guideline 220 (OECD 2004b), as described by Castro-Ferreira et al. (2012). The animals were obtained from cultures in the Department of Ecological Science, Vrije Universiteit, Amsterdam. Ten adults with visible eggs (white spots) in the clitellum region were selected and randomly assigned to a test jar. For exposure, 100 ml glass jars were used with approximately 23 g moist soil, with 5 replicates per treatment and control, covered with perforated aluminium foil to allow sufficient aeration. At the start and every week to each jar a few grains of dry baker’s yeast (Instant yeast from Algist Bruggeman N.V., Ghent, Belgium) was added for food. On day 21 of exposure, 10 ml ethanol (96%) was added to each test jar, and using tap water (100 ml) the contents of the jar were transferred into a 250 ml plastic container. To dye the worms, 200 µl of Bengal rose solution (1% ethanol) was added, and the container was covered with a plastic film and a lid. The sealed container was shaken vigorously for 10 s to ensure that the dye was spread evenly and stored overnight at 4 °C. In order to count the worms, the contents were first filtered through a 160 µm sieve using tap water, to break up and remove most of the soil particles. The remaining solution, containing the dyed worms (adults and juveniles) and sand material, was poured evenly over a white tray, pre-marked into fractions to enable efficient counting using a large movable magnifying glass and hand held counter.

The test with *Folsomia candida* followed OECD Guideline 232 (OECD 2009). Organisms were sampled from cultures of the Department of Ecological Science, Vrije Universiteit, Amsterdam. The animals used in the first test originated from arable land near Marknesse, The Netherlands, and had been cultured for over 20 years. Animals for the test with thiacloprid and the second test with imidacloprid originated from a culture kindly provided by the University of Aarhus, Denmark. A different culture had to be used to perform the second test due to problems with the animals from the Marknesse culture that occurred in the interval between the tests. Synchronization was developed in 125 ml translucent plastic containers filled with 2 cm deep substrate mixture of plaster of Paris and activated charcoal (8:1) moistened with distilled water. After synchronization, ten animals (10–12 days old) were carefully counted and transferred into 100 ml glass test jars with approximately 23 g moist test soil, with 5 replicates per treatment. At the start and every week a few grains of dry baker’s yeast (Instant yeast from Algist Bruggeman N.V, Ghent, Belgium) were added for food. The jars were covered with an opaque black plastic lid, loosely attached to the rim to allow for enough aeration. On day 28 of exposure, demineralised water was added to the test jars in order to transfer their content to a beaker, allowing the animals to float and to be photographed with a camera, model Nikon COOLPIX P510. These photographs were then analysed using ImageJ, a Java-based processing program adjusted for counting the animals.

*F. candida* avoidance test was carried out for 48 h using a method slightly adapted from ISO (2008), with 5 replicates per treatment. Clear circular plastic containers (4.5 cm wide × 3 cm deep) were used, with a clear plastic lid and adequate aeration. All containers were split into two sections—control and contaminated—with a piece of corrugated white plastic. Each side contained approximately 10 g of moist soil. After removal of the splitter, ten adults sampled from the laboratory culture were placed in the centre of the plastic container. After 48 h exposure, the splitter was reinserted and the animals on each side were counted by flotation. To test for the validity of the avoidance, in addition to treatment vs. control containers, dual controls with either water controls on both sides or water control and acetone control on either side were included.

*Oppia nitens* was tested following Princz et al. (2010), with an exposure of 35 days. Animals were taken from the cultures at the Department of Ecological Science, Vrije Universiteit, Amsterdam. *O. nitens* were not age synchronised, but light brown recently matured individuals were sampled exclusively, avoiding the white juveniles and mature dark-brown adults. Approximately 23 g moist soil was added to circular containers (4.5 cm wide × 3 cm deep), which had a plastic mesh base that was sealed with clear plastic wrap and placed in a plastic dish. Ten animals were added into the test container at random. Five replicates were prepared per treatment and control, and a few grains of baker’s yeast were added as a food source as required. On day 35 of exposure, the test containers were placed in a Tullgren apparatus for extraction of the mites. The plaster of Paris collecting jars were first saturated with water, avoiding mites’ desiccation during the extraction period, which was completed over 3-days employing a temperature of 30 °C at the top and of 5 °C at the bottom. Adults and juveniles (alive) were counted manually under a microscope.

Test procedures reported by Løkke and van Gestel (1998) were adapted for a 28-day sub-lethal toxicity test with *Porcellio scaber* using survival, weight change and feeding activity as the end points. Animals were sampled from Amsterdam Forest, The Netherlands, which is supposedly free of pesticides and metal contamination. To start exposures, individuals were weighed and divided into three weight classes: ≤20, 20–35 and 35–50 mg. Three animals, randomly selected from each of the three weight classes were transferred to each plastic jar (3 replicates per test concentration) with a diameter of 5 cm and a height of 3 cm filled with approximately 24 g moist soil and covered with a transparent plastic lid. Both imidacloprid and thiacloprid tests started at the same time. Weighed dried maple leaves cut into 1 × 1 cm pieces were added in the test jars as a food source. The jars were checked every 48 h for isopod survival and feeding activity. Remaining food was removed being replaced by fresh one, and water loss was replenished every 48 h. After 28 days the remaining live isopods were counted and weighed, and food consumption was determined.

All incubations took place in a climate room at 20 ± 2 °C at a photoperiod of 16:8 dark: light hours. All test jars were weighed at the start, so that water loss could be monitored on a weekly basis, and distilled water was added to maintain the level of moisture.

### Data analysis

Student T-test was applied to compare the results from the controls with and without acetone for each experiment. Since no significant differences were found for the tests (*p* value > 0.05), results of both controls were pooled for all data analyses.

LC_50_ was calculated using the trimmed Spearman-Karber method (Hamilton et al. 1977/1978). EC_50_ and EC_20_ values were estimated with the logistic dose response model of Haanstra et al. (1985). No observed effect concentration (NOEC) was estimated where possible using a one-way ANOVA followed by Dunnett’s post hoc test using SPSS 21. For the comparison of EC_50_ values obtained from the *F. candida* tests with water and acetone controls, a generalize likelihood ratio test was used (Sokal and Rohlf 1985).

The avoidance of *F. candida* was calculated as the percentage effect at each test concentration, comparing the number of animals in the test soil with that in the control as:

$$A = \frac{{C - T}}{N}$$

where A = avoidance (in %); C = number of animals in the control soil, T = number of animals in the test soil, *N* = total number of animals recovered.

Feeding activity of the isopods was calculated as grams of the dry leaf material consumed per grams of fresh mass, using the initial isopod weights, and it was tried to correct for mortality by taking into account the days on which a dead isopod was found. This corrected feeding activity was expressed as milligrams of leaf material consumed per gram of fresh isopod per day. Weight change of the surviving isopods was calculated relative to the initial mass.

## Results

Control performance of *E. andrei* met the validity criteria set by OECD (2004a). Adult survival decreased significantly at concentrations ≥1 mg/kg dry soil for imidacloprid and at 10 mg/kg dry soil for thiacloprid (Figure S1 in Supporting Information). LC_50_ was estimated at 0.77 mg/kg dry soil for imidacloprid and 7.1 mg/kg dry soil for thiacloprid (Table 1, Figure S1). There was no significant difference in biomass between the two controls (Student t-test; *p* = 0.989), and neither imidacloprid nor thiacloprid had a significant effect on earthworm biomass at concentrations where survival remained 100% (ANOVA; *p* > 0.05), but there was a 23% weight reduction at 3.3 mg/kg thiacloprid. Responses of *E. andrei* reproduction were similar for both neonicotinoids, as shown in Figure S2, with EC_50_s of 0.39 mg/kg dry soil for imidacloprid and 0.44 mg/kg dry soil for thiacloprid. The estimated EC_20_ was also similar for both neonicotinoids: 0.27 and 0.19 mg/kg dry soil, respectively (Table 1). NOEC was 0.3 mg/kg dry soil for both compounds.

**Table 1:** Summary of data on the toxicity (LC_50_, EC_50_, EC_20_, and NOEC) of imidacloprid and thiacloprid (mg/kg dry soil) on five selected soil organisms, exposed to both compounds in LUFA 2.2 soil. EC_50_ and NOEC values are for effects on reproduction, except for the isopods *Porcellio scaber* for which EC_50_ is based on food consumption and NOEC on weight change and food consumption

Species	Imidacloprid				Thiacloprid			
	LC_50_	EC_50_	EC_20_	NOEC	LC_50_	EC_50_	EC_20_	NOEC
*Eisenia andrei*	0.77 Δ	0.39	0.27	0.3	7.1 Δ	0.44	0.19	0.3
	(0.65–0.91)	(0.21–0.56)	(0.17–0.36)		(5.8–8.8)	(0.25–0.63)	(0.06–0.32)	
*Enchytraeus crypticus*	>30	2.0	1.2	1.0	>30	12	5.5	3.0
		(1.6–2.5)	(0.73–1.6)			(8.4–16)	(2.5–8.6)	
*Folsomia candida*								
Acetone^a^	0.20	0.097	0.045	0.1	–	–	–	–
	(0.14–0.25)	(0.040–0.16)	(0–0.09)					
Acetone^b^	0.62 Δ	0.30	0.17	0.25	2.7 Δ	2.4	1.3	1.1
	(0.53–0.73)	(0.20–0.39)	(0.11–0.30)		(2.1–3.5)	(1.5–3.2)	(0.53–2.1)	
Water^b^	0.47 Δ	0.26	0.17	0.1	3.9 Δ	1.7	0.95	1.1
	(0.38–0.58)	(0.20–0.32)	(0.10–0.24)		(3.2–4.9)	(0.97–2.4)	(0.37–1.5)	
*Oppia nitens*	360 Δ	119	na	100	>1000	76	na	>100
	(248–524)	(–)				(–)		
*Porcellio scaber*	7.6 Δ	6.7#		8.0#	>32	>32#		32#
	(5.2–11)	(–)		32*				16*

*na* not applicable, ∆ calculated using the Trimmed Spearman-Karber method (Hamilton et al. 1977), () confidence intervals, (–) no reliable confidence interval could be calculated

^a^ First test

^b^ Second test

#Effects on food consumption

*Effects on weight change

For enchytraeids, control performance met the criteria (OECD 2004b) with <20% mortality, >500 juveniles/jar and coefficient of variation for control reproduction being 28.5% for imidacloprid and 29.5% for thiacloprid. The highest concentration (30 mg/kg dry soil) reduced adult survival to 88% for imidacloprid and 68% for thiacloprid (data not shown). Because of the high survival rates no dose-response curve could be fitted to the data, and LC_50_ is considered to be >30 mg/kg dry soil for both insecticides (Table 1). Reproduction was significantly reduced by imidacloprid from ≥3 mg/kg dry soil and ≥10 mg/kg dry soil for thiacloprid (Figure S3), resulting in EC_50_s of 2.0 and 12 mg/kg dry soil, respectively (Table 1). EC_20_s were 1.2 mg/kg dry soil for imidacloprid and 5.5 mg/kg dry soil for thiacloprid (Table 1). NOEC was a factor of 3 times higher for thiacloprid (3.0 mg/kg dry soil) than for imidacloprid (1.0 mg/kg dry soil).

Control performance of *F. candida* in all reproduction toxicity tests was within the validity criteria with survival >80% (OECD 2009), although in the acetone control of the imidacloprid test juvenile numbers were somewhat lower than at the lower imidacloprid concentrations. These low control numbers, however, only slightly affected the estimated EC_50_ value, which was 0.30 mg/kg dry soil when based on all data and 0.26 mg/kg dry soil when omitting the controls. For the second test, the spiking method—acetone and water—did not show significant differences (*χ* ^2^_df=1_ = 0.65 for imidacloprid; *χ* ^2^_df=1_ = 1.06 for thiacloprid; n.s.) for both compounds (Figures S4 and S5). For imidacloprid, LC_50_ was 0.20 mg/kg dry soil for the first test and 0.47–0.62 mg/kg dry soil for the second test. For thiacloprid, the first test with acetone spiking failed. In the second test, LC_50_ was 2.7–3.9 mg/kg dry soil (Table 1, Figure S4). Reproduction of *F. candida* was affected by imidacloprid with EC_50_s of 0.097 mg/kg dry soil in the first test, and 0.26–0.30 mg/kg dry soil for the second test; EC_50_s for thiacloprid were 1.7–2.4 mg/kg dry soil (Table 1; Figure S5). EC_20_ for imidacloprid in the first test was 0.045 mg/kg dry soil and 0.17 mg/kg dry soil for the second test; for thiacloprid, EC_20_ was 0.95–1.3 mg/kg dry soil (Table 1). NOEC for effects on reproduction was 0.1–0.25 mg/kg dry soil for imidacloprid and 1.1 mg/kg dry soil for thiacloprid (Table 1).

The average avoidance or *F. candida* in the double control (solvent control on either side) was 40–60%, showing that the test conditions could be considered adequate for testing soil preferences. No dose-related significant avoidance or attraction was observed for the 48 h exposure with all avoidance below 70% as illustrated in Figure S6.

Mean *O. nitens* survival in the controls was 71–83%, with control reproduction being rather low especially in the thiacloprid test. Both survival and reproduction were dose-related reduced by imidacloprid (Figure S7) resulting in an estimated LC_50_ of 360 mg/kg dry soil and an EC_50_ of 119 mg/kg dry soil. Thiacloprid did not affect mite survival, but it had a significant effect on reproduction at 100 mg/kg dry soil (*p* = 0.006), with an EC_50_ of 76 mg/kg dry soil (Figure S7). As only two concentrations were tested (100 and 1000 mg/kg dry soil), confidence intervals were very wide and therefore not reliable (Table 1).

Control survival of *P. scaber* was 77% for water control and only 33% in the acetone control. Since survival was 80% at the lower test concentrations of both compounds, the water control was used for data analysis. Imidacloprid caused a dose-related decreased survival of the isopods, with an LC_50_ of 7.6 mg/kg dry soil. Thiacloprid did not show a clear dose-related decrease for survival, which was 56% at the highest test concentration, so LC_50_ is higher than the highest tested concentration (32 mg/kg dry soil) (Figure S8; Table 1). Weight change of the isopods decreased in a dose-related manner upon exposure to both compounds (Figure S9), but the effect was not significant for imidacloprid, probably due to the low number of individuals and large variation in data. Weight change was significantly affected by thiacloprid (one-way ANOVA; F = 3.21; df = 5; *p* = 0.034) but the data did not allow calculation of an EC_50_ (Table 1). Only imidacloprid significantly affected food consumption of the isopods (one-way ANOVA; F = 5.33; df = 6; p = 0.006), with an EC_50_ of 6.7 mg/kg dry soil (Figure S10; Table 1).

## Discussion

This study shows that: 1. *F. candida* and *E. andrei* were the most sensitive species to both neonicotinoids, enchytraeids and isopods showing intermediate sensitivity, and the oribatid mite *O. nitens* being relatively insensitive (Table 1); 2. Reproduction of both *F. candida* and *E. andrei* was significantly affected at imidacloprid concentrations within the range of the Predicted Environmental Concentrations (PEC) of 0.33–0.66 mg/kg dry soil (Mostert et al. 2002; Oi 1999); 3. Imidacloprid was more toxic than thiacloprid to both survival and reproduction, for all species tested.

Neonicotinoids are nervous toxins that act agonistically on the nicotinic acetylcholine receptors (nAChR) of insects. They bind to these receptors, mimicking acetylcholine, but are not degraded on the post-synaptic membrane by the acetylcholinesterase enzyme. As a result, the accumulation of neonicotinoids on the receptor can cause a collapse of the system due to a disruption of the nervous signals, leading to the death of the insect (Acda 2007; Matsuda et al. 2001; Tomizawa and Casida 2003). Different insect nAChR subunits have been identified—α (majority) and β—having a different sensitivity to different neonicotinoids (Thany et al. 2006). These subunits are, for instance, known to be responsible for the enhancement of the affinity for imidacloprid by the insect nAChR (Thany et al. 2006; Tomizawa and Casida 2003). Due to different chemical structures of the two neonicotinoids tested, it is likely that different subunits may be activated with distinctive effects on their toxicity (Matsuda et al. 2001; Thany et al. 2006).

Survival to reproduction ratio (SRR) (Table 2), as an alternative to the acute chronic ratio (ACR), is defined as ‘LC_50_: EC_50_’ and may be indicative of the specificity of the compound’s mode of action. According to Marinković et al. (2011) this ratio increases with the specificity of the action of the compound. For the earthworm *E. andrei*, SRR was much greater in response to thiacloprid than to imidacloprid, suggesting that thiacloprid has some specificity acting on sub-lethal endpoints leading to a reduction in the number of offspring. The low SRR value for imidacloprid is reflective of the high mortality rate of earthworms and reduced reproduction due to less surviving adults. Imidacloprid tests with earthworms described in the literature (Table 3) show LC_50_ values ranging from 2.8–25 mg/kg for formulations and 2.3–3.05 mg/kg for the pure compound, and EC_50_s of 0.9–4.1 mg/kg. This study found slightly higher toxicity of imidacloprid (pure compound) with an LC_50_ of 0.77 mg/kg dry soil and an EC_50_ of 0.39 mg/kg dry soil (Table 1), and this difference in toxicity could be due to the different type of soil used in these tests. Thiacloprid has not been tested very often and the literature reports LC_50_s of 2.68 and 10.96 mg/kg (pure compound) tested in OECD artificial soil (Table 3); our value of 7.1 mg/kg dry soil fits in this range, despite using LUFA 2.2. According to Pisa et al. (2015), insecticides can have a significant impact on animal metabolism, affecting the detoxification, intermediary and energetic metabolism pathways, and therefore reducing biomass gain. Other sublethal effects on earthworms are reduction in burrowing activity, which can have implications on feeding behaviour and biomass (Capowiez et al. 2010), and decreased cellulase activity (Luo et al. 1999) having consequences for food digestion. In this study, weight of the adult earthworms did not suffer any variation when exposed to imidacloprid. Animals exposed to thiacloprid, at a concentration of 3.3 mg/kg dry soil, lost around 23% of their weight, confirming the effect of thiacloprid on sublethal endpoints.

**Table 2:** Survival to Reproduction Ratio (SRR), defined as the ratio of LC_50_ and EC_50_ values for the toxicity of imidacloprid and thiacloprid to different species of soil invertebrates upon chronic exposure in LUFA 2.2 soil. See Table 1 for the LC_50_ and EC_50_ values

Species	Imidacloprid	Thiacloprid
*Eisenia andrei*	2.0	16
*Enchytraeus crypticus*	>15	>2.5
*Folsomia candida*	1.8–2.1	1.1–2.3
*Oppia nitens*	3.0	>13
*Porcellio scaber*	1.1	>1.0

**Table 3:** Literature data on the toxicity of imidacloprid and thiacloprid to soil invertebrates

Chemical	Species	LC_50_	EC_50_	Soil type/Properties	Compound/Formulation	Reference
Imidacloprid	*Eisenia fetida*	2.82 ^14d^	–	Artificial soil, 10% OM	Pure	Wang et al. (2012)
		3.05	0.95			Wang et al. (2015)
		2.3 ^14d^	–			Zang et al. (2000)
		2.3 ^14d^	–			Luo et al. (1999)
		25	–	Litter; 60% OM	Merit 75% a.i.	Kreutzweiser et al. (2008)
	*Eisenia andrei*	25.5	4.07	Tropical artificial soil; 10% OM	Gaucho 60% a.i.	Alves et al. (2013)
	*Dendrobaena octaedra*	5.7	–	Litter; 60% OM	Merit 75% a.i.	Kreutzweiser et al. (2008)
	*Aporrectodea nocturna*	3.74	–	Natural soil; 2.8% OM; pH 8.3	Confidor 20% a.i.	Capowiez et al. (2005)
	*Alollophobora icterica*	2.8	–			
	*Folsomia candida*	0.86^&^	0.26^&^	Artificial soil; 10% OM	Pure	Reynolds (2008)
		2.6^14d^	0.15	Artificial soil; 10% OM	Confidor 70% a.i.	Idinger (2002)
		0.25^@^				
		21^14d^	>1.0*	Tropical Artificial soil; 10% OM	Gaucho 60% a.i.	Alves et al. (2014)
		0.44	0.29	LUFA 2.2, 2,8% OM	Pure	Van Gestel et al. (2017)
Thiacloprid	*Eisenia fetida*	10.96^14d^	–	Artificial soil, 10% OM	Pure	Wang et al. (2012)
		2.68	0.2		Pure	Wang et al. (2015)
	*Eisenia andrei*	18.2 ^14d^	2.13 ^14d^	Artificial soil, 5% OM	Calypso 48% a.i.	Akeju (2014)
	*Enchytraeus crypticus*	25.6	5.6			
	*Folsomia candida*	4.38	2.1			
		9.0	1.5	LUFA 2.2; 2.8% OM	Pure	Van Gestel et al. (2017)
	*Hypoaspis aculeifer*	–	3674	Artificial soil, 5% OM	Calypso 48% a.i	Akeju (2014)

^*^Approx. 25% effect at highest test concentration of 1 mg/kg dry soil

^&^Values recalculated from original data derived from figures included in Reynolds (2008), using a logistic dose-response model; LC_50_ and EC_50_ values reported by Reynolds based on regression analysis were 1.38 and 0.60 mg/kg, respectively

^@^Recalculated from the data derived from Fig. 1 in Idinger (2002) using the Trimmed Spearman Karber method (Hamilton et al. 1977/1978)

When comparing the toxicity of imidacloprid and thiacloprid to *E. crypticus* to the toxicity they exerted on E*. andrei*, some rather unexpected differences were observed. These species belong to the same Phylum—Annelida, but despite this phylogenetic relation, they showed quite distinct sensitivity to the neonicotinoids, both for lethal and sublethal effects. Imidacloprid was a factor of >39 more toxic to *E. andrei* when considering effects on survival and a factor of 4–5 for effects on reproduction. For thiacloprid, the earthworms were a factor of >4 more sensitive than the enchytraeids when considering survival and a factor of 27–29 for reproduction (Table 1). The large difference in toxicity between the two Oligochaete species is likely due to their metabolism or differences in the abundance of high affinity sub units for neonicotinoids. SRR values for *E. crypticus* show a different scenario when compared with earthworms, suggesting a higher ratio for imidacloprid (Table 2), thus reflecting a specific mode of action of this neonicotinoid.

The SRRs for *F. candida* were almost the same for both neonicotinoids (Table 2), indicating that imidacloprid and thiacloprid might affect survival and reproduction in a similar way, which is an expected result since this species is closely related to insects enabling the compounds to act more efficiently on its nervous system. Nonetheless, imidacloprid is more toxic to *F. candida* than thiacloprid (Table 1), suggesting the existence of more high affinity imidacloprid subunits in this species. This difference in toxicity was confirmed by our findings in a study that ran in parallel with this one, and where we found quite similar LC50s and EC50s for the toxicity of imidacloprid and thiacloprid to *F. candida* in LUFA 2.2 soil (Van Gestel et al. 2017; Table 3), also confirming reproducibility of these data. The avoidance test on *F. candida* did not show avoidance behaviour within the 48 h of the experiment. This scenario was also observed by Alves (2010), when working with different compounds (imidacloprid, thiametoxam, carboxim + thiram and fipronil). It was expected that the insecticide would reduce the motility of the organisms and thus reduce their ability to avoid the contaminated soil.

The least sensitive species tested were *P. scaber* and *O. nitens* with the latter one being essentially tolerant to both neonicotinoids, even at concentrations above 1000 mg/kg soil (Table 1). Thiacloprid was more toxic than imidacloprid to reproduction than to survival of *O. nitens*, following the tendency seen for all species tested. The SRR ratios for *E. crypticus* and *O. nitens* (Table 2), however, have to be considered with caution, as the LC_50_ is above or close to the highest concentration tested. Akeju (2014), testing thiacloprid (Calypso) and acetamiprid (Epik) found that predatory mites (*Hypoaspis aculeifer*) had low sensitivity, with EC_10_ and EC_50_ values of 968 and 3674 mg/kg for thiacloprid and 447 and 651 mg/kg for acetamiprid, and Reynolds (2008) did not find any impact of imidacloprid (formulations) on the community (species richness, abundance or composition) of Acari (Oribatida and Mesostigmata Sub-orders) when compared with the control, supporting the results found on *O. nitens*. Szczepaniec et al. (2011, 2013) studied the impact of imidacloprid formulation on spider mite communities in order to verify reproduction outbreaks. They found that this phenomenon is related with the effects of this neonicotinoid on plants and predators, improving the palatability of the leaves by suppressing or reducing the production of defense hormones in plants and causing sub-lethal (impaired movement) or lethal effects on the predators of spider mites. The mites themselves appeared not to be influenced by the compound, neither when exposed by direct contact (through skin) nor by ingesting contaminated leafs. Conversely, the nervous system of the mites may be different from that of the insect target species, having n-AChR subunits with lower affinity for either imidacloprid or thiacloprid, requiring higher dosages of these compounds to produce any toxic effects. But this assumption of course needs further investigation.

Drobne et al. (2008) determined the toxicity of imidacloprid to *P. scaber* using dietary exposure and found that concentrations up to 50 mg/kg in food produced no lethal effect on juveniles or adults. The lowest concentration causing toxic effects was 10 mg/kg in food, affecting the feeding rate of adults and weight gain of juveniles. In our study, we found effects at lower concentrations, but this may be due to differences in the route of exposure. Earlier studies have shown that effect concentrations in soil may be lower than those in food, probably because of the lower availability of chemicals in the high organic food compared to the low organic soil (see e.g., Løkke and van Gestel 1998). The results of the study of Drobne et al. (2008) and our study do show that fitness of *P. scaber* could be at risk if imidacloprid accumulates in the leaf litter, given that they are macro-decomposers.

Different results for toxicity of imidacloprid and thiacloprid reported in the literature (Table 3) might be due to: the use of different types of soil with different contents of organic matter and different toxicity tests (acute or chronic). Cox et al. (1997, 1998) found that imidacloprid binds to soil organic matter in a reversible way, reducing its availability to soil organisms. Although the different modes of action of these neonicotinoids do not explain the difference in sensitivity among the animals tested, it helps understanding the possible effects these insecticides might have on non-target species. One other reason for the higher toxicity of imidacloprid could be its higher persistence in soil compared to thiacloprid. In a study running in parallel to this one, we determined imidacloprid and thiacloprid exposure concentrations in LUFA2.2 soil in a multi-generation experiment with *F. candida*. That study showed faster degradation of thiacloprid with estimated half-lives of 10–12 days, while imidacloprid showed little degradation and its half-life was estimated to be >125 days (Van Gestel et al. 2017).

## Conclusion

This study shows that the neonicotinoids thiacloprid and imidacloprid are highly toxic to soil invertebrates. Similar to studies on non-target pollinators, imidacloprid caused mortality at lower concentrations than thiacloprid. When comparing the sensitivity of species to both compounds *E. andrei* and *F. candida* are the most sensitive ones and *O. nitens* and *P. scaber* the most resistant, with SRR suggesting specificity of the mode of action of the neonicotinoids on different species. Long-term exposure of the tested species to these compounds can have important impacts on the soil ecosystem, with possible reduction of the offspring or the quality of the ecosystem services provided by these species. This may especially be the case for springtails and earthworms. The direct toxicity of neonicotinoids to non-target species warrants an evaluation of their long-term impact on agricultural soils and also the surrounding ecosystems.

## Electronic supplementary material

Supporting Information
