# Supplementary material for: Comparative toxicity of imidacloprid and thiacloprid to different species of soil invertebrates
Source: Ecotoxicology. 2017 Mar 23;26(4):555–64. doi: 10.1007/s10646-017-1790-7 (PMC5420372; doi:10.1007/s10646-017-1790-7)
Supplement: Supplementary file 1 — Supporting Information [file 10646_2017_1790_MOESM1_ESM.doc]

Supporting Information

To

**Comparative toxicity of imidacloprid and thiacloprid to different species of soil invertebrates**

Cláudia de Lima e Silva, Nicola Brennan, Jitske M. Brouwer, Daniël Commandeur, Rudo A. Verweij, Cornelis A.M. van Gestel*

Department of Ecological Science, Faculty of Earth and Life Sciences, Vrije Universiteit, De Boelelaan 1085, 1081 HV Amsterdam, The Netherlands

*Corresponding

e-mail: [kees.van.gestel@vu.nl](mailto:kees.van.gestel@vu.nl)

tel. +31-20-5987079

**Table S1:** Concentrations (mg/kg dry soil) of the two neonicotinoids used in the toxicity tests with five species of soil invertebrates.

| Species | Imidacloprid | Thiacloprid |
| --- | --- | --- |
| *Eisenia andrei* | 0-0.12-0.37-1.1-3.3-10 | 0-0.12-0.37-1.1-3.3-10 |
| *Enchytraeus crypticus* | 0-0.12-0.37-1.11-3.33-10-30 | 0-0.12-0.37-1.1-3.3-10-30 |
| *Folsomia candida* |  |  |
| First Test | 0-0.0014-0.0041-0.012-0.037-0.11-0.33-1.0 |  |
| Second Test | 0-0.031-0.063-0.13-0.25-0.5-1.0 | 0-0.014-0.041-0.12-0.37-1.1-3.3-10 |
| *Oppia nitens* | 0-100-1000 | 0-100-1000 |
| *Porcellio scaber* | 0-1-2-4-8-16-32 | 0-1-2-4-8-16-32 |


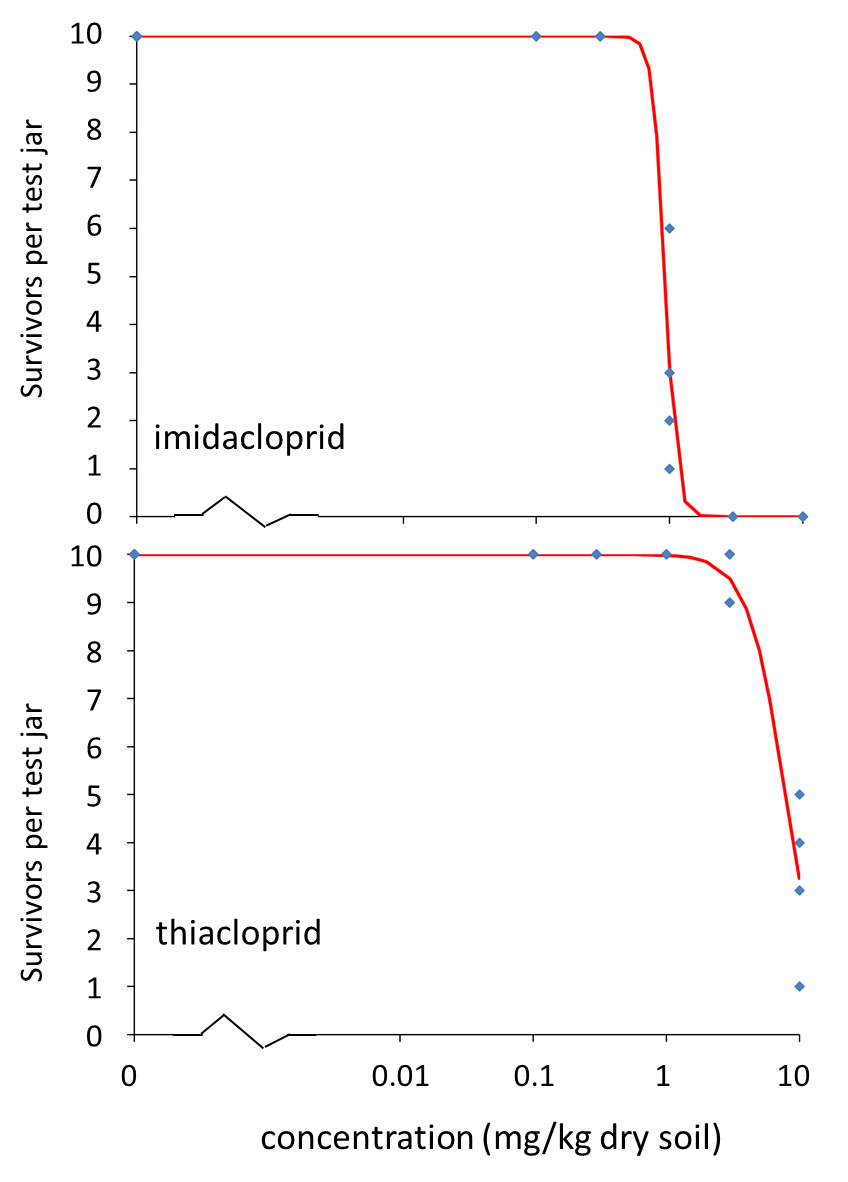


**Figure S1.** Effect of imidacloprid (top) and thiacloprid (bottom) on the survival of *Eisenia andrei* after 28 days exposure in LUFA 2.2 soil. Data points show the results of four replicate observations per test concentration, lines show the fit of a logistic dose-response model to the data.

**
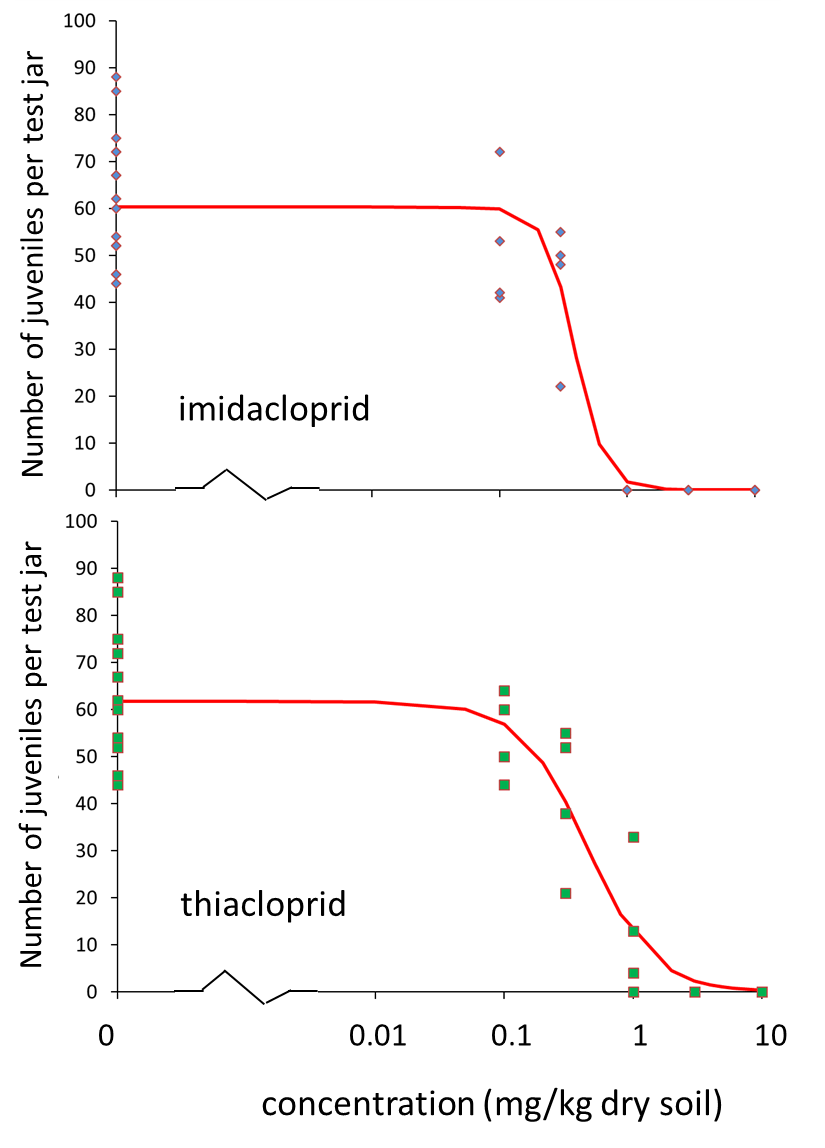
**

**Figure S2.** Effect of imidacloprid (top) and thiacloprid (bottom) on the reproduction of *Eisenia andrei* after 28 days exposure in LUFA 2.2 soil. The y-axis shows the number of juveniles recovered from each test jar after 56 days, 28 days after the adults were removed from the soil. Data points show the results of four replicate observations per test concentration, lines show the fit of a logistic dose-response model to the data.


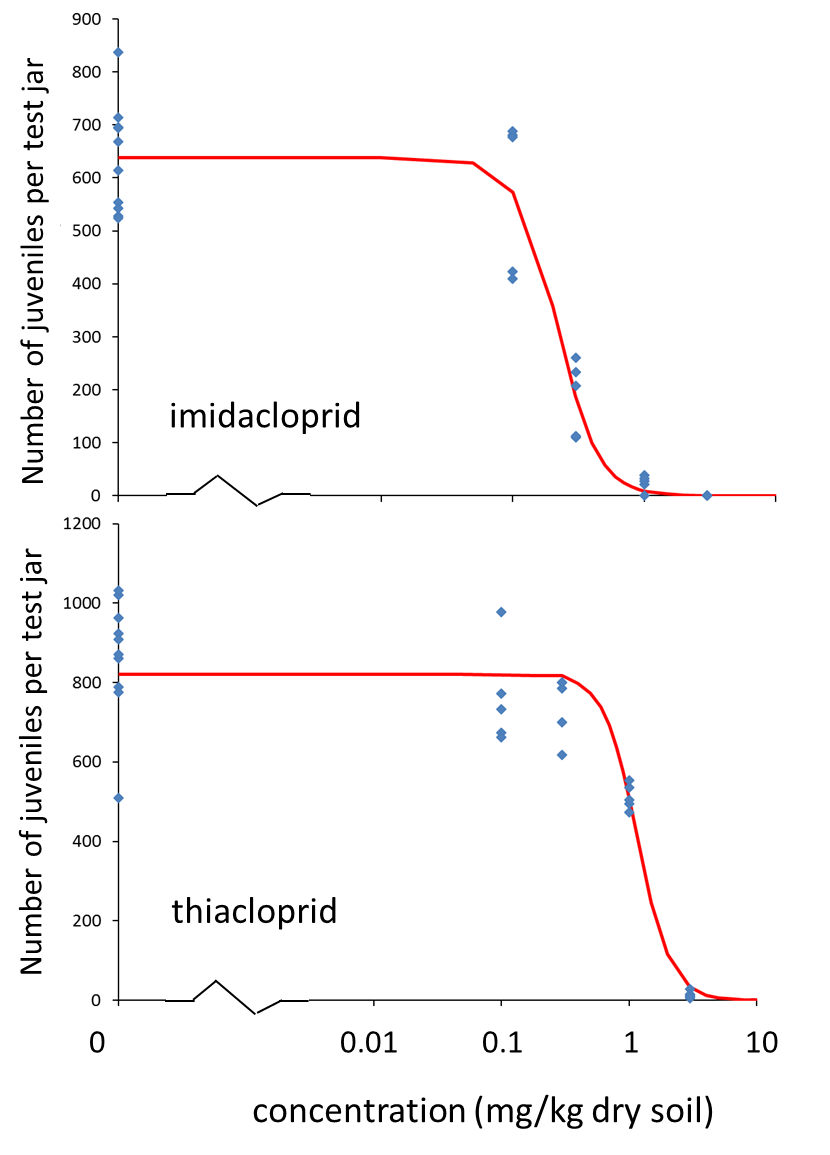


**Figure S3**. Effects of imidacloprid (top) and thiacloprid (bottom) on the reproduction of *Enchytraeus crypticus* after 21 days exposure in LUFA 2.2 soil. Data points show the results of five replicate observations per test concentration, lines show the fit of a logistic dose-response model to the data.


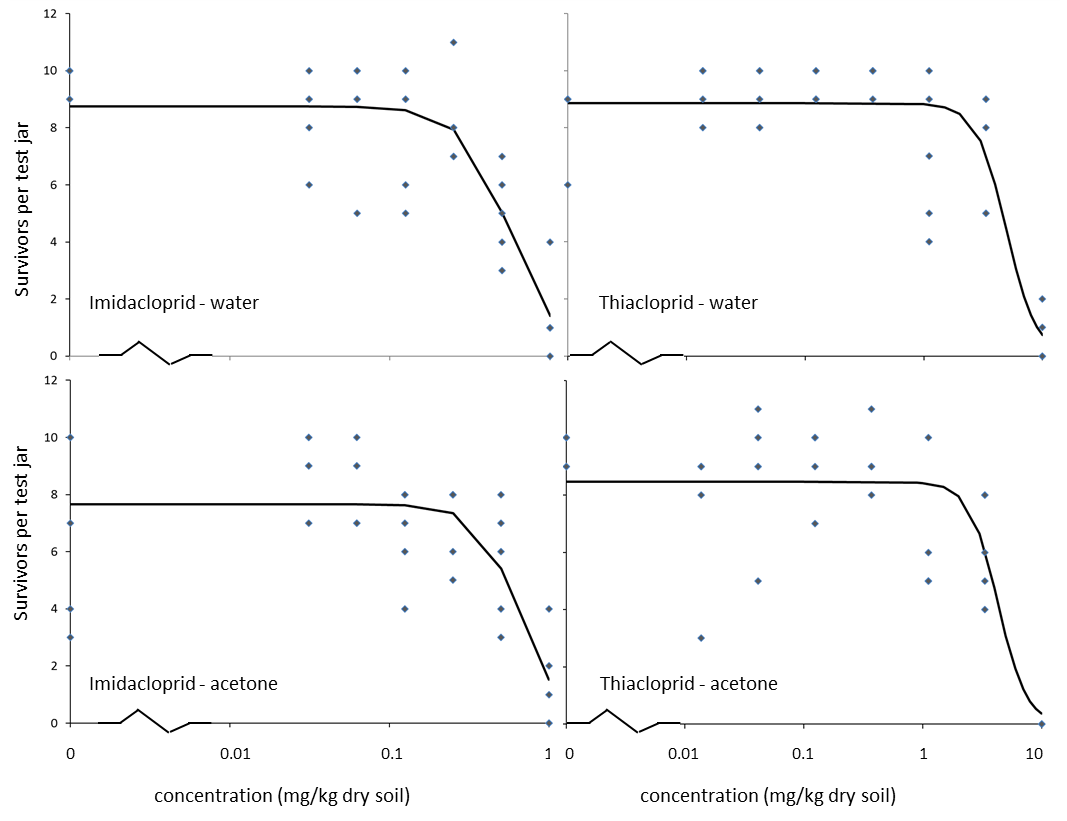


Figure S4: Effects of imidacloprid (left) and thiacloprid (right) on the survival of *Folsomia candida* after 28 days exposure in LUFA 2.2 soil. Data points show the results of five replicate observations per test concentration, lines show the fit of a logistic dose-response model to the data. Results are shown for tests using water (top) and acetone (bottom) to spike the soil.


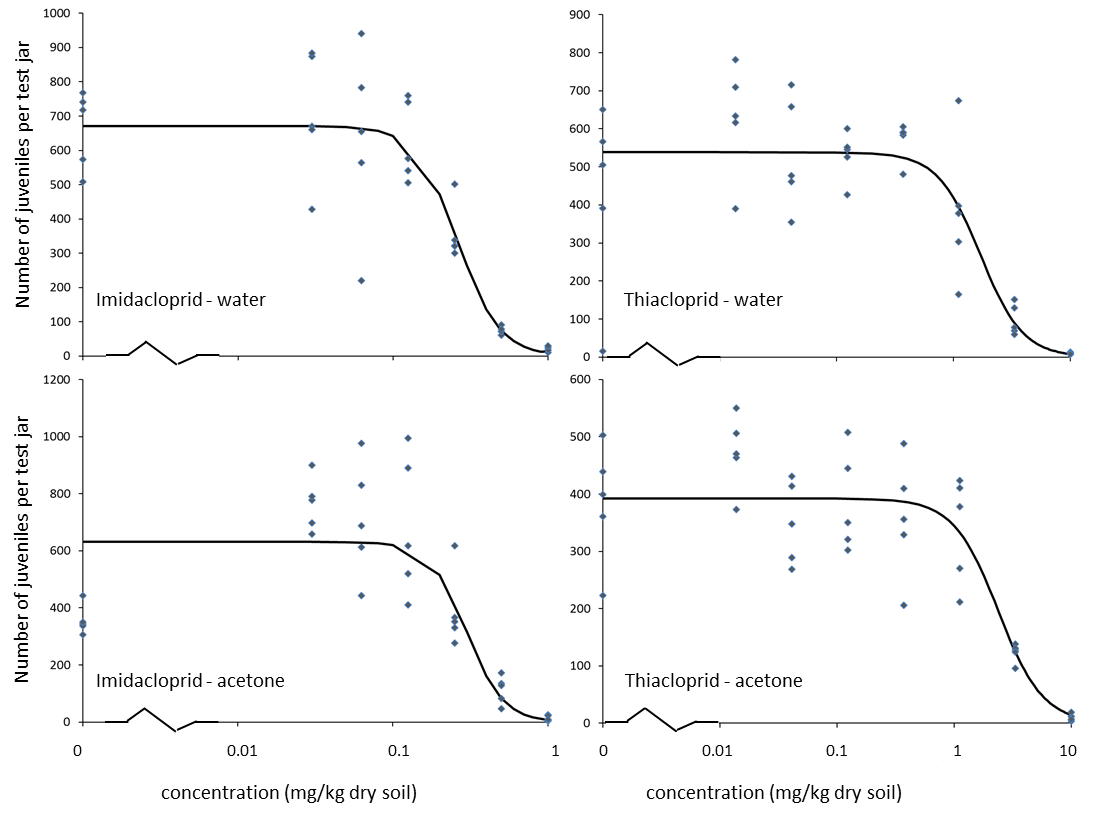


Figure S5: Effects of imidacloprid (left) and thiacloprid (right) on the reproduction of *Folsomia candida* after 28 days exposure in LUFA 2.2 soil. Results are shown for tests using water (top) and acetone (bottom) to spike the soil. Data points show the results of five replicate observations per test concentration, lines show the fit of a logistic dose-response model to the data.


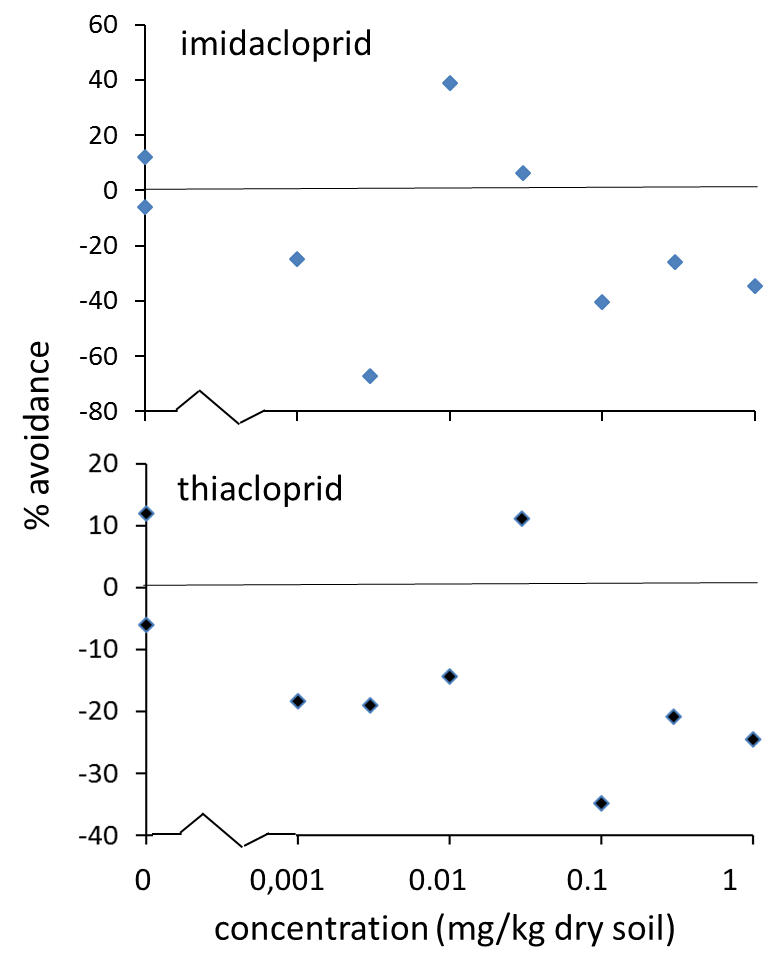


**Figure S6.** The average avoidance response of *Folsomia candida* when exposed for 48 hours to imidacloprid (top) or thiacloprid (bottom) in LUFA 2.2. The % avoidance was calculated as described in the main text. Nominal concentrations are given in mg/kg dry soil.


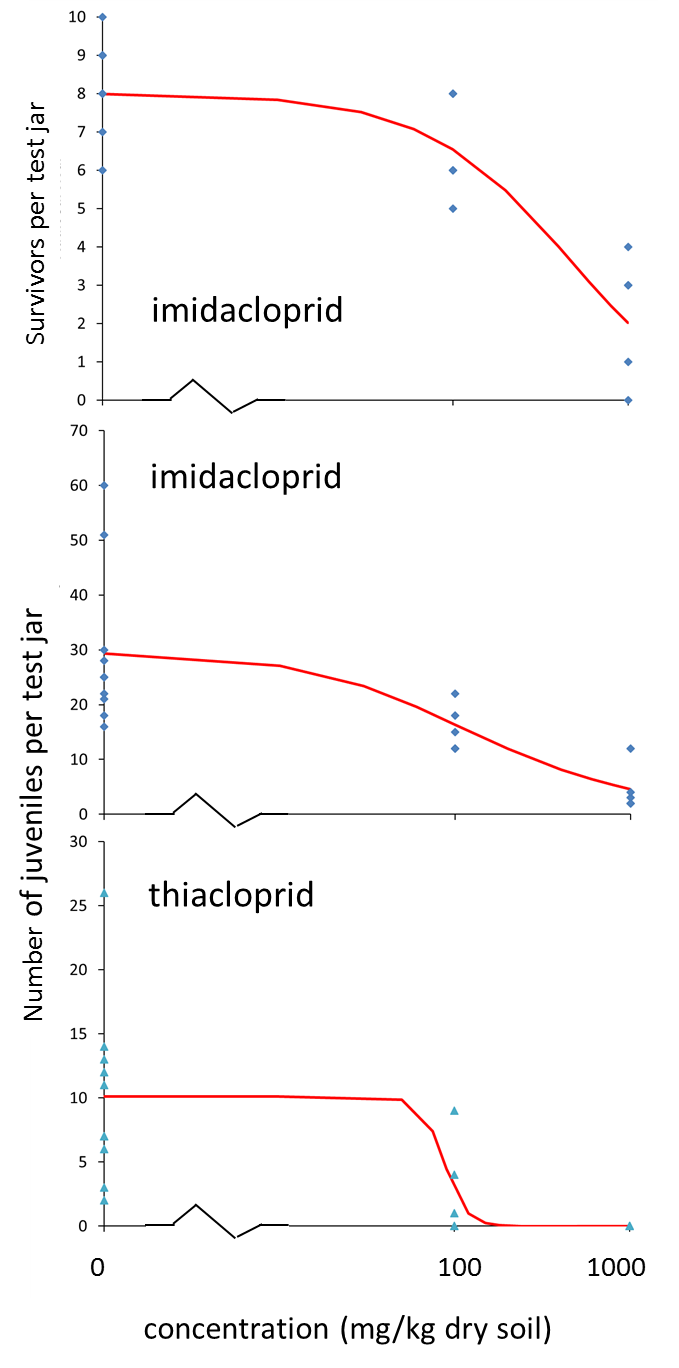


**Figure S7**. Effects of imidacloprid on the survival (top) and of imidacloprid (middle) and thiacloprid (bottom) on the reproduction of *Oppia nitens* after 35 days exposure in LUFA 2.2 soil. Data points show the results of 5 replicate observations per test concentration, lines show the fit of a logistic dose-response model to the data.


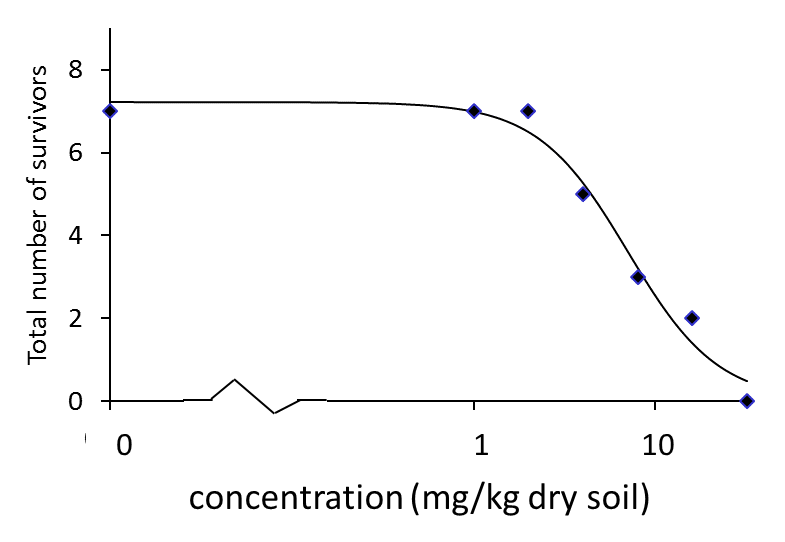


**Figure S8:** Effect of imidacloprid on the survival of *Porcellio scaber* after 28 days exposure in LUFA 2.2 soil. Data points show the summed response of 3 replicate observations, with 3 animals each, per test concentration. Line shows the fit of a logistic dose-response model to the data.


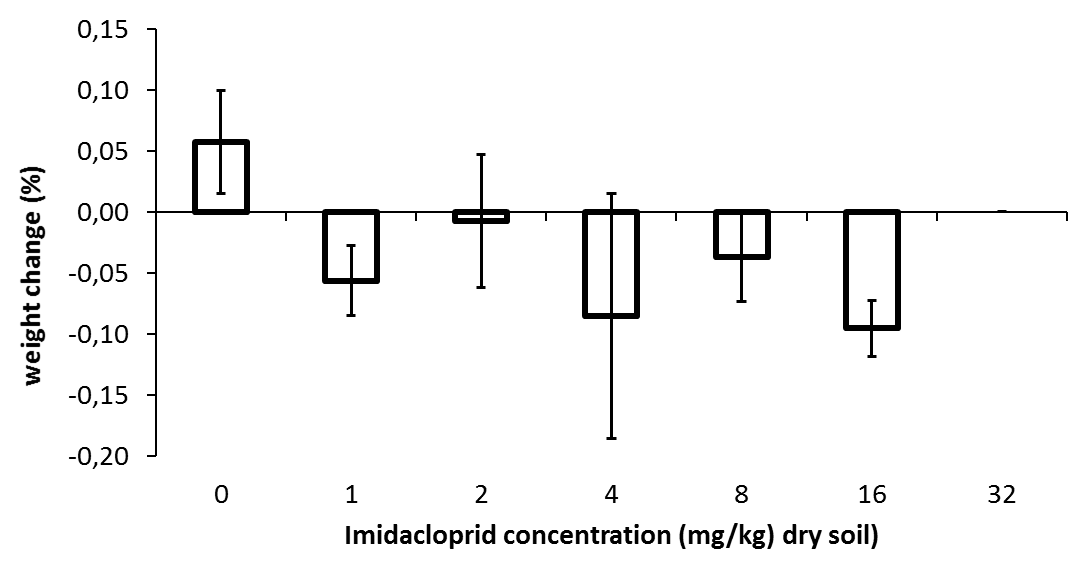

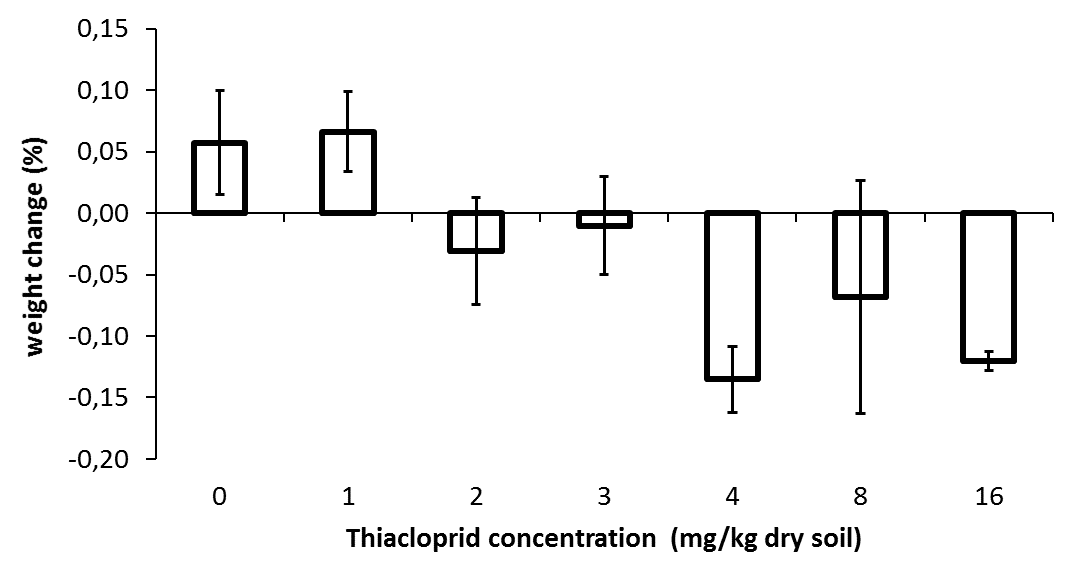


**Figure S9:** Effect of imidacloprid (top) and thiacloprid (bottom) on the weight change of *Porcellio scaber* after 28 days exposure in LUFA 2.2 soil. Weight change is expressed in % of the initial mass. The error bars show the Standard Error.


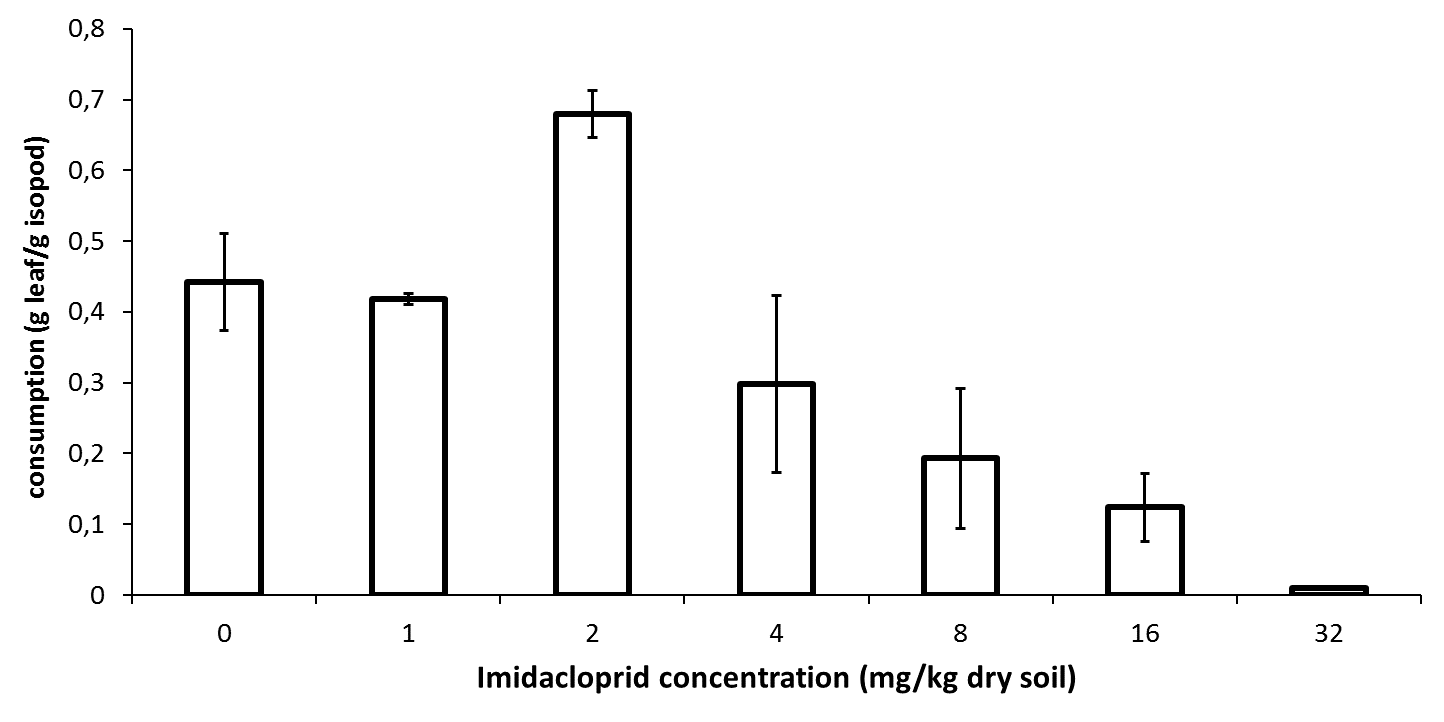

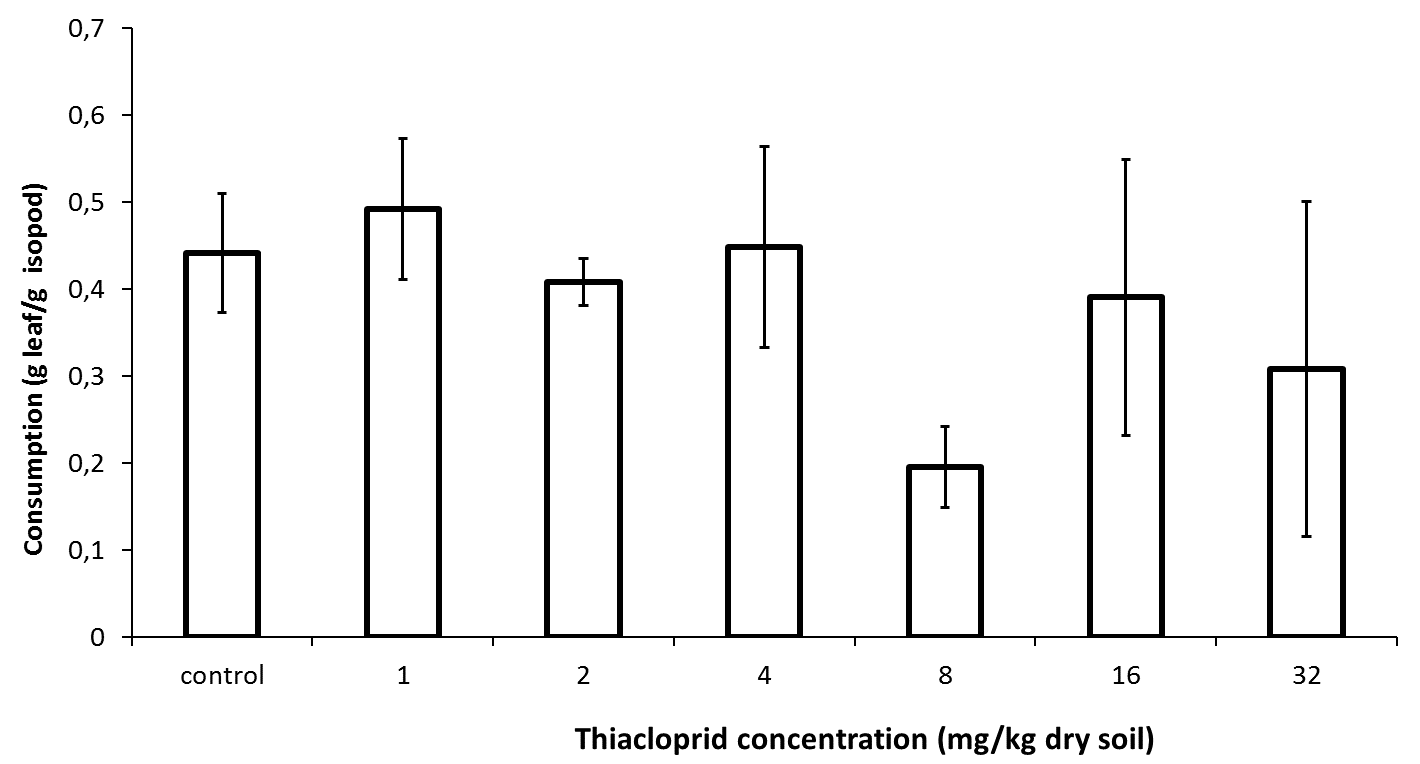


**Figure S10:** Effect of imidacloprid (top) and thiacloprid (bottom) on the food consumption of *Porcellio scaber* after 28 days exposure in LUFA 2.2 soil. The error bars show the Standard Error.
